# *Pristionchus uniformis*, should I stay or should I go? Recent host range expansion in a European nematode

**DOI:** 10.1002/ece3.28

**Published:** 2011-12

**Authors:** Isabella D'Anna, Ralf J Sommer

**Affiliations:** Department of Evolutionary Biology, Max Planck Institute for Developmental BiologySpemannstrasse 37, Tübingen D-72076, Germany

**Keywords:** Biogeography, host range expansion, host-switching, *Pristionchus uniformis*, species invasion

## Abstract

*Pristionchus pacificus* has been developed as a model system in evolutionary developmental biology, evolutionary ecology, and population genetics. This species has a well-known ecological association with scarab beetles. Generally, *Pristionchus* nematodes have a necromenic association with their beetle hosts. Arrested dauer larvae invade the insect and wait for the host's death to resume development. Only one *Pristionchus* species is known to frequently associate with a non-scarab beetle. *Pristionchus uniformis* has been isolated from the chrysomelid *Leptinotarsa decemlineata*, also known as the Colorado potato beetle, in Europe and North America, but is also found on scarab beetles. This unusual pattern of association with two unrelated groups of beetles on two continents requires the involvement of geographical and host range expansion events. Here, we characterized a collection of 81 *P. uniformis* isolates from North America and Europe and from both scarab beetles and *L. decemlineata*. We used population genetic and phylogenetic analyses of the mitochondrial gene *nd2* to reconstruct the genetic history of *P. uniformis* and its beetle association. Olfactory tests on beetles chemical extracts showed that *P. uniformis* has a unique chemoattractive profile toward its beetle hosts. Our results provide evidence for host range expansion through host-switching events in Europe where *P. uniformis* was originally associated with scarab beetles and the nematode's subsequent invasion of North America.

## Introduction

The expansion of the host or the geographic range of an organism can be favored by host-switching events (Secord and Kareiva 1996). Host-switching is defined as the horizontal transfer from one host to another and represents a process that has attracted increasing consideration in ecology and evolutionary biology in recent times (Ricklefs and Fallon 2002; Page 2003; Zarlenga et al. 2006). Host-switching by parasites refers to colonization of “foreign” host species in which it did not occur previously (Clayton et al. 2003), although host-switching as a concept is not restricted to parasites. One process that often involves host-switching events is species invasions (Sax et al. 2005). The invasion of a host can favor host-switching for two reasons. First, an invasive host can carry microorganisms that can infest new hosts. Second, an invasive host species can be infected by new microorganisms in the invaded area. There is a growing awareness on species invasion that turned a major threat to native biological diversity, such as that associated with trading and tourism. Species invasion and host-switching often result in the extinction of native organisms, particularly on islands, making these processes tremendously important for the sustainability of biodiversity (Sax et al. 2005).

**Figure 1:**
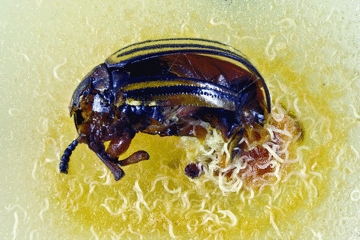
Worms resume development on beetle carcass. *Pristionchus uniformis* adult nematodes feeding on a *Leptinotarsa decemlineata* carcass. Image courtesy of Andreas M. Weller.

Nematodes are ubiquitous, mostly small animals that have successfully invaded marine, freshwater, soil, and parasitic habitats. Several nematode species have been developed as important model systems in biology, including *Caenorhabditis elegans*, which is one of the best-studied model organisms in modern biology (The *C. elegans* Research Community 2011) and *Pristionchus pacificus*, a model organism in evolutionary biology and ecology (Hong and Sommer 2006a; Herrmann et al. 2007, 2010; Zauner et al. 2007; Brown et al. 2011). Field studies revealed that *Pristionchus* nematodes have a necromenic association with their beetle hosts, in such a case, arrested dauer larvae invade the insect, wait for the host to die naturally, and resume development by feeding on growing microorganisms on the carcass (Herrmann et al. 2006a; Weller et al. 2010; Bento et al. 2010) (Fig. 1). More than 20 *Pristionchus* species have been identified in world-wide samplings in association with scarab beetles, whereas other beetle and insect groups (with two exceptions, see below) do not harbor *Pristionchus* nematodes on a regular basis (Herrmann et al. 2006a, b, 2007, 2010; Weller et al. 2010). The phylogeny of *Pristionchus* nematodes has been studied by detailed molecular investigations indicating the existence of three clades, a European, a North American, and a basal Asian clade (Mayer et al. 2007, 2009). Several *Pristionchus*–beetle associations have been characterized, such as the oriental beetle (*Exomala orientalis*) with *P. pacificus*, the cockchafer (*Melolontha melolontha*) with *P. maupasi*, and dung beetles (*Geotrupes* spp.) associated with *P. entomophagus* (Herrmann et al. 2006a).

So far, only one *Pristionchus* species could also be frequently recovered from a non-scarab beetle. *Pristionchus uniformis* has been isolated from the chrysomelid *Leptinotarsa decemlineata*, also known as the Colorado potato beetle, in Europe and North America (Herrmann et al. 2006a, b). Interestingly, *P. uniformis* also shows an association with scarab beetles on both continents. The gonochoristic species *P. uniformis* is unique in the genus *Pristionchus* for two reasons. First, it has stable associations on two continents with members of two ecologically disparate families of beetles. Second, while scarab beetles are commonly associated with numerous nematode species, *L. decemlineata* does not harbor other nematodes suggesting that *P. uniformis* has evolved specific traits that allow the identification, infestation, and survival on this chrysomelid beetle.

A potential correlation between host-switching and species invasion also exists in *P. uniformis.* While most *Pristionchus* species are restricted to single continents, *P. uniformis* is one of three species in this genus that was found on several continents (Herrmann et al. 2006a, b). *Pristionchus pacificus* is a true cosmopolitan species that associated with different scarabs on different continents (Herrmann et al. 2010), *P. entomophagus* is a species that is found in association with multiple insects (Herrmann et al. 2010; Herrmann and Sommer, pers. comm.), whereas *P. uniformis* is the only species of that genus that is frequently found on two groups of beetles. Interestingly, one of the beetle hosts of *P. uniformis*, *L. decemlineata*, is an example of a biological invasion from the United States to Europe (Balachowsky 1963). Given the *P. uniformis*–*L. decemlineata* association, it is therefore tempting to speculate that their invasions into Europe might be correlated. However, phylogenetic analysis of *Pristionchus* nematodes clearly indicates that *P. uniformis* is part of the European clade of the genus and not the North American clade (Mayer et al. 2007). Therefore, a simple coinvasion of nematode and beetle seems unlikely.

Despite the growing awareness of species invasion and host-switching, little is known about the genetic conditions and the population genetic structures associated with these processes. The major economic pest of potato crops, *L. decemlineata*, is an interesting exception, as recent molecular studies indicate that European *L. decemlineata* populations contain only a small fraction of the genetic variability known from North America (Grapputo et al. 2005).

Here, we describe a population genetic study on an invasive nematode. The aim of this study was to analyze the directionality of both biological invasion and host-switch during geographic and host range expansion events. Based on a collection of 81 *P. uniformis* isolates from North America and Europe, both from scarab beetles and *L. decemlineata*, we used population genetic and phylogenetic analyses of the mitochondrial gene *nd2* to reconstruct the genetic history of the obligate outbreeding species *P. uniformis* and its beetle association. Our results provide evidence for host-switching events in Europe where *P. uniformis* was first associated with scarab beetles. Population structure of European *P. uniformis* strains show a much higher genetic diversity than American strains arguing for an invasion from Europe to North America.

## Materials and Methods

### Nematode sampling and breeding conditions

Strains used in this study are the result of various collections from diverse localities (see Table S1). Nematodes were isolated from insects collected during field trips or sent to our Institute from other laboratories. Some strains were isolated from soil samples. The nematode lines used in the study were obtained using the standard procedure to isolate *Pristionchus* nematode from the field (Herrmann et al. 2006a). The insect or the soil samples were transferred to the laboratory and placed on nematode growth medium (NGM) agar plate. The insects were sacrificed by cutting them in half. Using a dissecting scope, the plates were checked daily over a period of 1–3 weeks for emerging and reproducing nematodes. From the emerging nematodes, we established isofemale lines: gravid females were transferred to new plates to establish laboratory lines. For taxonomic determination, morphological and molecular methods were used (see next paragraph). For breeding and maintenance of *P. uniformis*, we followed standard culture methods described previously for *C. elegans* (Brenner 1974). Stock maintenance, fecundity, and generation time tests were done on NGM agar plates with *Escherichia coli* OP50 lawns and kept at 20°C. Mating experiments for species identification and fecundity tests between *P. uniformis* strains were performed on plates with one virgin female together with five males.

### Biological properties of *P. uniformis*

To study the population genetics of *P. uniformis* and the potential patterns of species invasion and host-switching, we sampled a total of 81 isolates of *P. uniformis* in different locations in Europe and North America. Twenty-one of these strains have been collected from different scarab beetles, 32 from *L. decemlineata*, whereas the remaining strains were obtained from various sources including soil and rotten plants (Table S1). To study the biology of *P. uniformis*, we have first observed if major difference in the life-history traits was detectable among the different 81 strains. We checked brood size, generation time, and sex ratio between strains from scarab beetles, from *L. decemlineata*, isolated in Europe and North America, performing crossing in plates with one virgin female together with five males.

### Hypotheses to be tested

**Figure 2:**
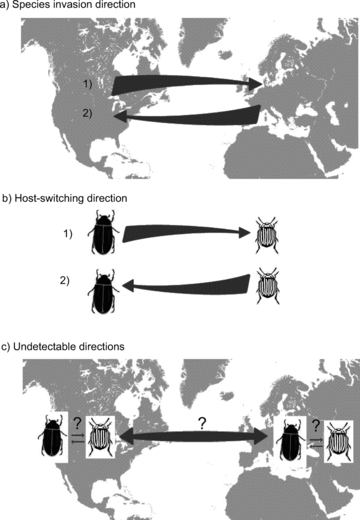
*Pristionchus uniformis* geographical and host origin hypotheses. *Pristionchus uniformis* possible migration direction, from North America toward Western Europe (A1) or vice-versa (A2). (B) Primary *P. uniformis* host association and following host-switch hypotheses: from scarab spp. to *Leptinotarsa decemlineata* (B1) or vice-versa (B2). (C) When no clear *P. uniformis* genetic structure is found, neither species invasion nor host-switch direction can be detected. Big arrows size is proportional to the *P. uniformis* population genetic diversity. Darker cartoon represents a scarab spp. host and the striped cartoon represents the *L. decemlineata*. World map modified after Graphic Factory CC.

In *P. uniformis*, we can test hypotheses about geographic and host range expansion. Different hypotheses are possible. Concerning how species invasion might have occurred, *P. uniformis* could have been primarily present in North America and subsequently invaded Europe (Fig. 2A1) or vice-versa (Fig. 2A2). A European origin of *P. uniformis* is supported by the observation that this species is part of a European clade of *Pristionchus* species (Mayer et al. 2007). In the context of *P. uniformis* beetle association origin, we could also test the direction of the host-switching between the two major host, scarabs and *L. decemlineata* (Fig. 2B). Under both of these circumstances (Fig. 2A and B), invasion and host-switching, the genetic variability in *P. uniformis* isolates obtained from the receiver location and host might have resulted in a reduction, similar to what has been described for the *L. decemlineata* (Grapputo et al. 2005). A final, alternative scenario would be that no genetic structure exists between material from different beetle hosts and geographic origins due to multiple independent switches and invasions (Fig. 2C).

### Sequencing

For species identification, DNA was prepared from single individual nematodes per strain, and species identity was assessed by their having identical small subunit ribosomal RNA (SSU) sequences as described in Herrmann et al. (2006a). To evaluate the genetic variability between *P. uniformis* isolates, by conducting BLAST searches of the *P. pacificus* mitochondrial sequence to an early version of the *P. entomophagus* genome, a species closely related to *P. uniformis* (Mayer et al. 2009), we then obtained the mitochondrial genes for *nd2* and *cyt b*. The following polymerase chain reaction (PCR) primers were designed: IS12109 CGCAAAAGATATACGCCAAT and IS12120 TTCTCCCAAAGGAACTTTACC. The *nd2* mitochondrial gene fragment of 789 bp was cloned and sequenced from both ends in all 81 *P. uniformis* strains used in this study. Genomic DNA was prepared from three overgrown 6-cm NGM plates. Plates were washed three times in distilled water. DNA was isolated with a genomic DNA extraction kit (MasterPure™ DNA Purification from Epicentre Biotechnologies, Madison, WI, USA). The DNA was diluted to approximate 25 ng/µl for the PCRs. PCR was performed in 25-µl 1× PCR buffer (Amersham Biosciences [currently GE Healthcare Europe Gmbh], Munich, Germany) containing 1 U *Taq* DNA Polymerase (Amersham Biosciences [currently GE Healthcare Europe Gmbh], Munich, Germany), 0.5 µM of each primer, 0.2 mM of each deoxynucleotide triphosphate, and 4 µl of DNA lysate. PCR experiments were performed as follows: initial denaturation at 94°C for 2 min, followed by 35 cycles of denaturation at 94°C for 30 sec, primer annealing at 50°C for 30 sec, and extension at 72°C for 60 sec. A final incubation step at 72°C was performed for 7 min. PCR products were diluted 1:20 with ddH_2_O and sequenced without further purification from both ends (Big Dye terminator protocol, Applied Biosystems, CA, USA, ABI373*xl* capillary platform).

### Data analyses

Sequence trace files were visualized with the software SeqMan (DNAStar Inc.) and aligned with Bioedit version 7.0. 5. 3; (Hall 1999). The sequences have been deposited in GenBank and can be retrieved by their accession code JN555657-JN555737 (*P. uniformis*) and JN562471 (*P. maupasi*). For phylogenetic analyses, the best-fit substitution model and the parameter settings were determined in Find Best-Fit Substitution Model (ML) in MEGA version 5 (Tamura et al. 2011) using the Akaike information criterion (Akaike 1974; Posada and Crandall 1998). The parameters for the selected model HKY+I+Γ were as follows: estimated base frequencies: A = 0.331, C = 0.081, G = 0.109, T = 0.479; proportion of invariable sites (I) = 0.40. Phylogenetic relationship among the *P. uniformis* strains was assessed based on 789 bp of the *nd2* mitochondrial gene with in MEGA 5 (Tamura et al. 2011), and heuristic search using maximum likelihood (ML) as optimality criterion. The bootstrap consensus tree was inferred from 10.000 replicates. The tree was edited using Dendroscope (Huson et al. 2007). Divergence parameters, values for neutrality tests, and haplotype data were obtained with the program DNASP version 5.10.01, (Librado and Rozas 2009). The population parameter θ was calculated from the number of segregating sites. Haplotypes variation output file, with parsim informative sites highlighted, was generated with MEGA 5 (Tamura et al. 2011). Haplotypes distribution among host and *F*_ST_ statistics were calculated with Arlequin version 3.5. 1. 2; (Excoffier et al. 2005). Network analyses of the mitochondrial sequences were performed with Network version 4.6.0.0 available at fluxus-engineering.com (Bandelt et al. 1999).

### Beetle extracts and chemotaxis assays

The attraction of *Pristionchus* nematodes toward specific compounds in chemotaxis assays might recapitulate their behavioral response in nature toward their preferred host (Hong and Sommer 2006b). Chemical extracts from the insect host of *P. uniformis* were used to investigate attraction profiles of two *P. uniformis* strains. Feeding adult males of the scarab *Phyllophaga anxia* were collected with pheromone traps in Geneva (NY), USA in May 2007. Pupae of *L. decemlineata* were derived from our laboratory culture that was initiated in 2006 from a group of beetles collected in Tübingen (Germany). For the cuticular hydrocarbon extract, three *P. anxia* adults and 25 *L. decemlineata* pupae were placed in glass sample tubes. The beetles were then soaked in dichloromethane (CH_2_Cl_2_) for 24 h at 23°C. The washes were then vacuum dried at 30°C in small glass sample tubes, then resuspended in 150 µl of pure ethanol. Dichloromethane without a beetle specimen but processed the same way served as a counter-attractant control. Chemotaxis assays were performed on 8.5-cm Ø NGM agar plates, as previously described for *Pristionchus* species (Hong and Sommer 2006b). The host odorants have been tested on two *P. uniformis* strains (RS5167 and RS5303) and for comparison on *P. pacificus* (PS312). Mixed stage nematodes containing mostly adults were washed three times in M9 buffer and then loaded onto the agar plates, which had been prepared with two point sources of odors. As attractant, the host extract and control a solvent and sodium azide to anesthetize the nematodes on opposite sides of each plate. The chemotaxis index is defined as [the number of worms at the attractant site – worms at control site]/total number of worms scored. At least two separate experiments were conducted for each strain, and each experiment consisted of 6–10 replicates. On average, each replicate represented the outcome for 30–100 worms. Only the highest chemotaxis index was recorded, which peaked between 15 and 16 h at 23°C. Two-tailed two-sample Student's *t*-test was done in Microsoft Excel.

## Results

### Life-history traits variation in *P. uniformis*

First, we analyzed the biological properties of 11 representative strains of *P. uniformis* (the reference strain and isolates from different host association and with different geographic origin) to evaluate if different host associations were correlated with differences in life-history traits. No obvious differences among *P. uniformis* isolates were found. For example, the sex ratio was close to 50% males in all strains. Similarly, the generation time was between 3 and 4 days and fecundity was between 91 and 170 (Table S2), which is typical for the genus *Pristionchus*. These findings suggest that the different host association of *P. uniformis* did not result in adaptive differences in life-history traits.

### Phylogenetic and network analyses for *P. uniformis*

**Figure 3:**
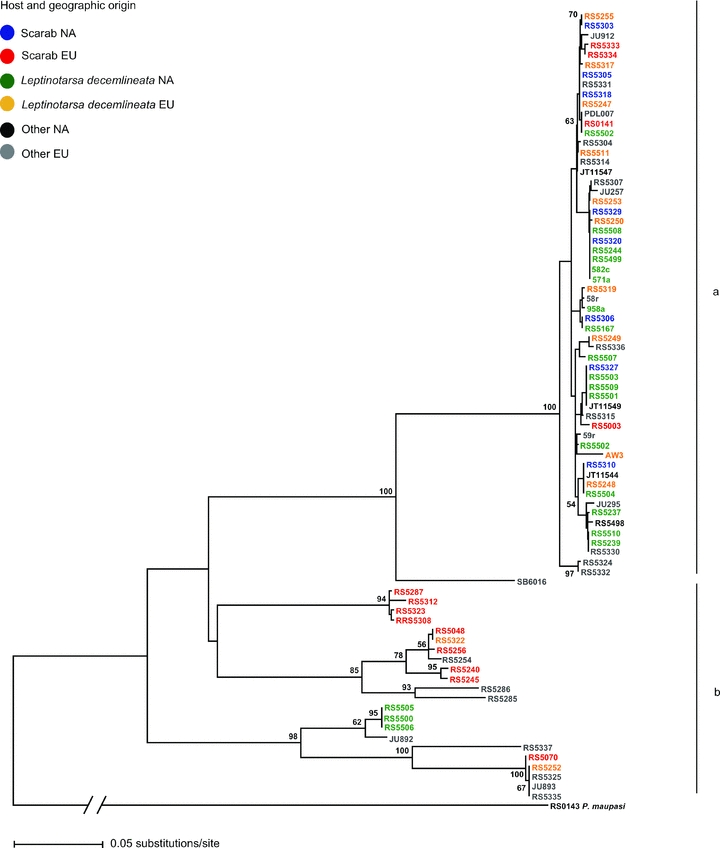
Phylogenetic relationship of 81 *Pristionchus uniformis* strains. The maximum likelihood (ML) tree was reconstructed from aligned mitochondrial gene *nd2* sequences. Robustness of the tree topology was evaluated by 10.000 ML replications. The support values are shown at the nodes. Branch lengths are proportional to genetic divergence. Geographical origin (EU = European; NA = North America) and host association are color coded at the taxon label. Strains with “other” origin were isolated from soil or non-scarab or non-*Leptinotarsa decemlineata* beetles. Letters indicate geographical subgrouping.

To determine the genetic variance of *P. uniformis*, we analyzed all strains by comparing the rapidly evolving mitochondrial marker *nd2*. A total of 789 bp were compared among the 81 strains isolated from various localities, sources, and hosts. The *nd2* sequences were used to construct a phylogeny based on the genotypes of these 81 strains. As outgroup, the sequence of *P. maupasi* RS0143 was included. Figure 3 shows a rooted, ML tree of the phylogenetic relationship between the mitochondrial *nd2* gene genotypes of *P. uniformis* isolates. The majority of the strains fall into a derived monophyletic group of strains that are genetically very similar to each other. In this clade, there is no clear separation between strains associated with scarab or chrysomelid beetles (Fig. 3A). Similarly, strains from North America and Europe are interspersed, and several *P. uniformis* strains with identical haplotypes, such as RS5303 and RS5255, were found on scarab beetles and *L. decemlineata* in the United States and Germany, respectively (Fig. 3A). Similarly, multiple *P. uniformis* strains, that is, RS5287, RS5312, RS5323, and RS5308, as well as RS5048, RS5256, RS5245, and RS5240 were collected from the same site, but are genetically unrelated (Fig. 3B). The major findings of the phylogenetic analysis can be summarized as follows. First, North American strains always share a clade with European strains. Second, there are three clades formed by European strains only. Third, there are no deeper clades and there is no support for grouping the clades together. Taken together, these data suggest a European origin of *P. uniformis* and multiple colonization events from Europe to North America. Also, no population genetic structure that would completely dissociate hosts and localities can be detected.

**Figure 4:**
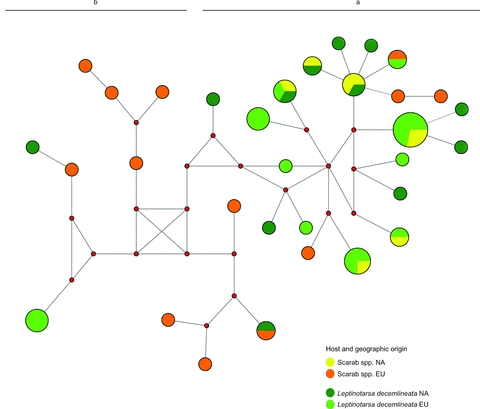
Median joining network for a selection of 53 mitochondrial haplotypes of the *nd2* gene amplified in *Pristionchus uniformis* strains collected in scarab spp. or *Leptinotarsa decemlineata*. Circles represent unique haplotypes. The areas of the circles are proportional to the number of samples sharing each haplotype. Different colors represent different geographic origin (EU = European; NA = North American) and host associations as indicated on the figure. Letters indicate subgroups detected also in Figure 3.

In Figure 4, the *nd2* gene network defines 32 haplotypes in a selection of 53 strains isolated from scarab spp. and *L. decemlineata.* Each haplotype is restricted to one of the four predefined groupings (*L. decemlineata* from Europe; *L. decemlineata* from North America; scarab from Europe; scarab from North America) except for eight haplotypes that are shared between the two beetle groups and three cases also between continents (Fig. 4A; Table S3). Genetic diversity of scarab derived *P. uniformis* (π = 0.102) was nearly twice that of the *L. decemlineata* derived strains (π = 0.056) (Table 2). Additionally, the estimation of the genetic differentiation between these two host association subgroups of *P. uniformis* resulted to be significant (*F*_ST_ = 0.091 ± 0.000, *P*-value = 0.000).

**Table 2:** Divergence estimates in geographic subgroups based on the mitochondrial gene *nd2*

	*n*	*S*	*H*	*H*d	π	θ	Tajima's D
*P. uniformis*							
Total	81	319	49	0.979	0.083	0.105	–0.515
Europe	49	316	41	0.991	0.105	0.117	–0.515
North America	32	156	14	0.915	0.038	0.055	–0.923
*P. pacificus*							
Total	22	126	19	0.983	0.037	0.049	–0.605
North America	7	28	5	0.857	0.013	0.015	–0.474
Europe, Asia, La Réunion	12	133	12	1.000	0.046	0.053	–0.207

S = number of segregating sites, H = number of haplotypes, Hd = haplotype diversity, π = nucleotide diversity, θ = level of polymorphisms from S, Tajima's D test for the assumption of neutral sequence selection evolution. *Pristionchus uniformis* sequence length = 789 bp and *P. pacificus* length = 777 bp. Data for *P. pacificus* are from Zauner et al. 2007 and Molnar et al. 2011.

### Population structure for *P. uniformis*

The deep sampling of *P. uniformis* and the availability of type material from Europe and North America make it possible to study the ancestral origin of this species. The *nd2* gene analysis suggests that the *P. uniformis* strains collected in Europe are more genetically diverse than the strains from North America. As mentioned in the previous paragraph, North American *P. uniformis* are always found in clades together with European strains. In contrast, European strains are found in all major clades covering a higher sequence divergence and occupying more basal positions in the rooted phylogeny with three clades formed by European sequences only.

Only one group of American isolates, RS5505, RS5500, and RS5506, has a more basal position in the phylogeny (Fig. 3B). This group is clearly distinct from the other American strains, supporting the hypothesis of multiple expansions from Europe to North America.

The European origin of *P. uniformis* is confirmed by considering the nucleotide diversity found between *P. uniformis* strains from North American and Europe. Specifically, North American strains show a lower nucleotide diversity (π) of 0.04, whereas the European strains show a π of 0.10 (Table 2). The nucleotide diversity of *P. uniformis* is substantially higher than the diversity of related hermaphroditic species (Tables 2 and S4). Specifically, the comparison of *P. pacificus* strains from North America, Europe, and La Réunion in the Indian Ocean revealed diversity values that were a factor of two to three times lower than those observed for *P. uniformis* (Table 2). Furthermore, the estimated genetic differentiation (*F*_ST_) was significant between the two geographic subgroups of *P. uniformis* (Europe vs. North America) (*F*_ST_ = 0.103 ± 0.000; *P*-value = 0.000).

### P. *uniformis* host recognition

**Figure 5:**
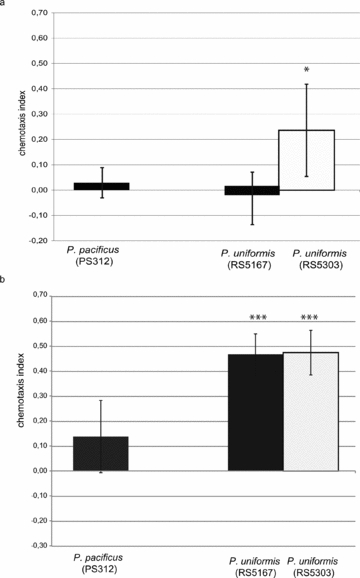
Chemoattraction assay of *Pristionchus uniformis* strains compared to *P. pacificus*. (A) Nematode attraction toward dichloromethane extraction of *Phyllophaga anxia* adults. *Significant difference between *P. uniformis* (RS5167), *Leptinotarsa decemlineata* derived, and (RS5303) *P. anxia* derived, *P* < 0.05 by two-sampled *t*-test. (B) Nematode attraction toward dichloromethane extraction of *Leptinotarsa decemlineata* pupae When compared to *P. pacificus* (PS312), both *P*. *uniformis* strains (RS5167 and RS5303) are significantly more attracted towards the *L. decemlineata* extract, ***P < 0.001 by two-samples *t*-test. Error bars denote 95% confidence intervals and each bar represents 10–15 replicates.

Previous studies found that *Pristionchus* species associated with different beetles have distinct chemotaxis profiles toward insect compounds (Hong and Sommer 2006b). To test whether *P. uniformis* strains show specificity in their host recognition, we performed chemotaxis experiments on two American *P. uniformis* strains, one isolated from *L. decemlineata* (RS5167) and one from *P. anxia* (RS5303). Both of these strains fall genetically in the derived clade A (Fig. 3A). Chemotaxis profiles obtained from exposing nematodes to cuticular extracts of the two beetles provide three important observations. First, the *P. anxia* derived strain RS5303 shows a stronger attraction to *P. anxia* beetle extract than *L. decemlineata* derived RS5167 (*t*-test, *P*-value = 0.018) (Fig. 5A). Second, RS5303 and RS5167 show similar attraction to washes of the “novel” beetle host *L. decemlineata* and both are significantly more attracted than *P. pacificus*, which has never been found associated with any of the test-hosts (Fig. 5B) (*t*-test, *P*-value = 0.000). These results suggest that, first, chemoattraction mechanisms evolve rapidly, and second, that some *P. uniformis* strains have lost the ability to recognize certain scarab beetles as potential hosts.

## Discussion

Growing interest in invasion biology has mirrored the escalation of species invasion. (Elton 1958; Richardson and Pyšek 2007). While species invasions are often a problem in agriculture, recent studies also focus on the basic biogeography of species invasion to gain insight into the factors and processes that control diversity and distribution at different scales (Richardson and Pyšek 2007). Here, we investigated the biogeography of the nematode *P. uniformis* that has been found tightly linked to one of the most famous insect invaders of Europe, the Colorado potato beetle (*L. decemlineata*).

Nematode associations with other organisms are common and numerous entomophilic nematodes have associations with their hosts, ranging from loose phoresy to strict species-specific parasitism (Sudhaus 2008). Many insect hosts spend most parts of their life cycle in habitats that facilitate nematode attachment. For example, scarab beetle females usually deposit eggs in the soil and grubs feed on roots often for several years, for example, the American May beetle *P. anxia* and the European cockchafer *M. melolontha*. The beetle *L. decemlineata* has a different and much shorter life cycle approximately 30 days long. After hatching from eggs on potato leaves, larval instars feed on leaves, only entering the soil during the last instar to pupate. Thus, scarab beetles and *L. decemlineata* are in the soil where they can get into contact with nematodes, during overlapping but distinct parts of their life cycles. However, a quantitative assessment of the soil–beetle exchange of *Pristionchus* nematodes awaits further study, which may be considerably advanced by involving transgenic technology, available for *Pristionchus* nematodes, for monitoring the movement of nematodes.

Numerous studies on parasite–host associations have described a horizontal transfer from one host to another, defined as host-switching (Page 2003). The switch to a new host is considered one of six types of events that are commonly found in host–parasite evolution (Page 2003). A host-switch involves an initial “expansion” of the parasite's host range and often, the parasite persists on the original host. Successful “colonization” of foreign hosts requires that the parasite “disperse” to that host and is able to “establish” a viable breeding population on it.

Our results revealed that Europe is the likely native area of *P. uniformis* because genetic diversity is greater in Europe than in North America. Thus, *P. uniformis* most likely *expanded* to a new continent, namely from Europe to North America and succeeded in *colonizing* a “new” insect host, the Colorado potato beetle (Table 1). We found eight cases, in which a mitochondrial haplotype is shared between the two hosts, supporting the hypothesis that *P. uniformis* can successfully associate and reproduce on both beetle groups (Fig. 4; Table S3). Assuming that identical mitochondrial sequences did not originate independently in different hosts or locations by parallel (or convergent) mutations, these findings provide evidence for host-switching during recent *P. uniformis* evolution.

**Table 1:** Divergence estimates in host-associated subgroups based on the mitochondrial gene *nd2*

*P. uniformis* (length = 789 bp)						
	*n*	*S*	*H*	*H*d	π	θ
Total	53	278	32	0.969	0.078	0.097
Scarab spp.	21	250	19	0.990	0.102	0.109
*Leptinotarsa decemlineata*	32	241	21	0.960	0.056	0.093

S = number of segregating sites, H = number of haplotypes, Hd = haplotype diversity, π = nucleotide diversity, θ = level of polymorphisms from S.

Our analysis of a collection of 81 *P. uniformis* genotypes from North America and Europe favors scenario 2 of the potential biological invasions offered in Figure 2A. We provide clear evidences for a European origin of *P. uniformis* based on the basal positions of European clades and much higher genetic diversity of strains found in Europe (Table 2). Colonization has probably happened at least twice, apparent from a clade of North American nematodes (RS5505, RS5500, RS5506) distinct from that of the highly related clade (Fig. 3B). This finding is supported by the phylogenetic position of *P. uniformis* in the European group of species within the genus *Pristionchus* (Herrmann et al. 2007; Mayer et al. 2007). Given the recent invasion of *L. decemlineata* to Europe, host-switching event might have occurred in Europe. Under these circumstances, *P. uniformis* most likely has invaded North America from Europe. However, it remains unknown if *P. uniformis* invaded North America in association with a host beetle or by another way of transportation. Also, our results do not allow to exclude the potential scenario that *P. uniformis* has been introduced to North America prior to the invasion of Europe by *L. decemlineata.* Data presented herein, provide the first molecular support to characterize the complexity of species invasion and host-switching events of a *Pristionchus* nematode. The ease with which these nematodes can be isolated and characterized might make them a useful system to further investigate the biogeography of insect associated nematodes.

So far *P. uniformis* is the only *Pristionchus* species that is found consistently on disparate families of hosts among more than 20 species collected worldwide (Mayer et al. 2009). These observations suggest that host-switching is exceptional in *Pristionchus* evolution and might require special genetic features. The adaptation to the new ecological niche requires a new host-seeking behavior, special chemoattractive properties, and novel survival attributes. Among these adaptations, chemoattraction can be studied under laboratory conditions by using chemoattraction assays. We were able to show that *P. uniformis* has a species-specific preference for its beetle hosts’ odor profile. *Pristionchus uniformis* significantly diverged from the model species *P. pacificus* in its chemoattraction profiles toward the extract of the scarab *P. anxia* and the chrysomelid *L. decemlineata*. Interestingly, we observed also an intraspecific discrepancy in chemotaxis when testing the two *P. uniformis* strains on *P. anxia* extracts. The *P. anxia* odor was attractive only for the *P. anxia* derived *P. uniformis* strain. These findings suggest that chemoattraction mechanisms can evolve rapidly, and that some *P. uniformis* strains have lost the ability to recognize certain scarab beetles as potential hosts. It is important to note that this finding might be influenced by the high diversity of scarab beetles. In particular in North America, the group of scarab beetles is very diverse (Arnett et al. 2002) and investigations about the species specificity of *Pristionchus* with these North American scarab beetles await future analysis.

One other, crucial factor for successful host-switching is the ability to overcome competitive exclusion by other residents of the new host (Barker 1994). Interestingly, the “new”*P. uniformis* host *L. decemlineata* is well protected against predators by the secretions of defensive glands (Dalzoe et al. 1986) and by the toxic substances present in the haemolymph (Hsiao and Fraenkel 1969). Indeed, *L. decemlineata* is poor in associated nematodes, most likely because toxic substances prevent a successful invasion by nematodes. Therefore, the switch of *P. uniformis* toward *L. decemlineata* was not influenced by competition from resident nematodes and might have been favored by low predation rates. At the same time, *P. uniformis* must have evolved a mechanism to overcome the toxicity associated with the *L. decemlineata* habitat. However, we have so far been unable to identify such mechanisms; our investigations of the *L. decemlineata* haemolymph did not show any evidence for resistant mechanisms of *P. uniformis*, when compared to other nematode species.

## Supporting Information

**Table S1.** *Pristionchus uniformis* strains used in the study.

**Table S2.** Comparison of life-history traits among representative strains.

**Table S3.** *nd2* mitochondrial gene haplotype variation and distribution among hosts.

**Table S4.** Divergence estimates of mitochondrial genes indifferent *Pristionchus* species in *cyt b*.
