# Supplementary material for: Pristionchus uniformis, should I stay or should I go? Recent host range expansion in a European nematode
Source: Ecol Evol. 2011 Dec;1(4):468–78. doi: 10.1002/ece3.28 (PMC3287333; doi:10.1002/ece3.28)
Supplement: Supplementary file 1 [file ece30001-0468-SD1.doc]

**Supplementary material**

| **Supplementary table 1** *Pristionchus uniformis* strains used in the study | | | |
| --- | --- | --- | --- |
| Strain | Host taxon/soil | sampling location | collector |
| 571a | *Leptinotarsa decemlineata* (Chrysomelidae) | US (East), Ohio | C.Hoy |
| 58 r | soil | UK, Scotland | R.Rae |
| 582c | *Leptinotarsa decemlineata* (Chrysomelidae) | US (East), Ohio | C.Hoy |
| 59 r | soil | UK, Scotland | R.Rae |
| 681b | *Leptinotarsa decemlineata* (Chrysomelidae) | US (East), Freyburg (ME) | A.Miller |
| 958a | *Leptinotarsa decemlineata* (Chrysomelidae) | US (East), Bridgewater, (ME) | A.Miller |
| AW3 | *Leptinotarsa decemlineata* (Chrysomelidae) | Germany (south), Tübingen | A.Weller |
| JT11544 | rotten tomato | US, (west) Samamish, (WA) | J. Madeoy |
| JT11547 | rotten apple | US, (west) Samamish, (WA) | B. Kraemer |
| JT11549 | rotten squash | US, (west) Samamish, (WA) | J.H. Thomas |
| JU257 | soil | France (north), Etrechy (Paris) | E. Pierre |
| JU295 | soil | France (north), Valle de la Viosne (Paris) | M.A.Felix |
| JU892 | compost heap | Germany (south), Tübingen | R.J. Sommer |
| JU893 | *Geophilus* sp*.* (Chilopoda) | France (south), Lagorce (Nimes) | M.A.Felix |
| JU912 | vineyard soil, rotten grape | France (centre), Le Blanc, (Poitiers) | M.A.Felix |
| PDL007 | soil | Belgium, Landegem (Nevele) | P. De Lay |
| RS0141 | *Melolontha melolontha* (Scarabaeidae) | Germany (north-east),Menz, (Berlin) | W. Sudhaus |
| RS5003 | *Melolontha melolontha* (Scarabaeidae) | Germany (south-west), Obergrombach | n.d |
| RS5048 | *Amphimallon* sp. (Scarabaeidae) | Serbia, Novi Sad (Beograd) | A.Weller |
| RS5070 | *Melolontha melolontha* (Scarabaeidae) | Germany (north-east), Usedom | M.Herrmann |
| RS5167 | *Leptinotarsa decemlineata* (Chrysomelidae) | US (East), New York | n.d |
| RS5237 | *Leptinotarsa decemlineata* (Chrysomelidae) | US (north-east), Ohio | M.Herrmann |
| RS5239 | *Leptinotarsa decemlineata* (Chrysomelidae) | US (north-east), Ohio | M.Herrmann |
| RS5240 | *Rhizotrogus* sp*.* (Scarabaeidae) | Serbia, Novi Sad (Beograd) | A.Weller |
| RS5244 | *Leptinotarsa decemlineata* (Chrysomelidae) | US (north-east), Ohio | M.Herrmann |
| RS5245 | *Amphimallon* sp*.* (Scarabaeidae) | Serbia, Novi Sad (Beograd) | A.Weller |
| RS5247 | *Leptinotarsa decemlineata* (Chrysomelidae) | Germany (south), Tübingen | n.d |
| RS5248 | *Leptinotarsa decemlineata* (Chrysomelidae) | Germany (south), Tübingen | C.Lanz |
| RS5249 | *Leptinotarsa decemlineata* (Chrysomelidae) | Germany (south), Ammerbuch (Tübingen) | M.Herrmann |
| RS5250 | *Leptinotarsa decemlineata* (Chrysomelidae) | Germany (south), Ammerbuch (Tübingen) | M.Herrmann |
| RS5252 | *Leptinotarsa decemlineata* (Chrysomelidae) | Germany (south), Ammerbuch (Tübingen) | M.Herrmann |
| RS5253 | *Leptinotarsa decemlineata* (Chrysomelidae) | Germany (south), Tübingen | n.d |
| RS5254 | soil | Montenegro, Budva (Kosmack) | M.Grbic |
| RS5255 | *Leptinotarsa decemlineata* (Chrysomelidae) | Germany (south), Tübingen | n.d |
| RS5256 | *Amphimallon* sp. (Scarabaeidae) | Serbia, Novi Sad (Beograd) | A.Weller |
| **(continued) Supplementary table 1** *Pristionchus uniformis* strains used in the study | | | |
| Strain | Host taxon/soil | sampling location | collector |
| RS5285 | *Carabidae* sp. | Bulgaria (north), Tabachka (north Sofija) | M.Herrmann |
| RS5286 | *Staphylinidae* sp. | Bulgaria (north), Tabachka (north Sofija) | M.Herrmann |
| RS5287 | *Phyllopertha* sp.(Scarabaeidae) | Germany (south), Tuttlingen (black forest) | A.Weller |
| RS5303 | *Phyllophaga anxia* (Scarabaeidae) | US (north-east) Geneva, (NY) | I.D´Anna |
| RS5304 | rotten potato | Germany (south), Heilbronn | M.Herrmann |
| RS5305 | *Phyllophaga anxia* (Scarabaeidae) | US (north-east) Geneva, (NY) | I.D´Anna |
| RS5306 | *Phyllophaga futilis* (Scarabaeidae) | US (north-east) Geneva, (NY) | I.D´Anna |
| RS5307 | rotten potato | Germany (south), Heilbronn | M.Herrmann |
| RS5308 | *Phyllopertha* (Scarabaeidae) | Germany (south), Tuttlingen (black forest) | A.Weller |
| RS5310 | *Phyllophaga anxia* (Scarabaeidae) | US (north-east) Geneva, (NY) | I.D´Anna |
| RS5312 | *Phyllopertha* sp. (Scarabaeidae) | Germany (south), Tuttlingen (black forest) | A.Weller |
| RS5314 | *Carabidae* sp. | Germany (south), Waiblingen (Stuttgart) | M.Mueller |
| RS5315 | rotten potato | Germany (south), Heilbronn | M.Herrmann |
| RS5317 | *Leptinotarsa decemlineata* (Chrysomelidae) | Germany (south), Ammerbuch (Tübingen) | M.Herrmann |
| RS5318 | *Phyllophaga anxia* (Scarabaeidae) | US (north-east) Geneva, (NY) | I.D´Anna |
| RS5319 | *Leptinotarsa decemlineata* (Chrysomelidae) | Germany (south), Tübingen | n.d |
| RS5320 | *Phyllophaga anxia* (Scarabaeidae) | US (north-east) Geneva, (NY) | I.D´Anna |
| RS5322 | *Leptinotarsa decemlineata* (Chrysomelidae) | Germany (south), Tübingen | n.d |
| RS5323 | *Phyllopertha* sp. (Scarabaeidae) | Germany (south), Tuttlingen (black forest) | A.Weller |
| RS5324 | rotten potato | Germany (south), Tübingen | C.Lanz |
| RS5325 | rotten potato | Germany (south), Tübingen | C.Lanz |
| RS5327 | *Phyllophaga anxia* (Scarabaeidae) | US (north-east) Geneva, (NY) | I.D´Anna |
| RS5329 | *Phyllophaga anxia* (Scarabaeidae) | US (north-east) Geneva, (NY) | I.D´Anna |
| RS5330 | beach soil | France (south), Port Bregançon (cote d'Azur) | I.D´Anna |
| RS5331 | rotten potato | Germany (south), Tübingen | C.Lanz |
| RS5332 | rotten potato | Germany (south), Tübingen | C.Lanz |
| RS5333 | *Geotrupes* sp. (Scarabaeidae) | Corsica | M.Herrmann |
| RS5334 | *Geotrupes* sp. (Scarabaeidae) | Corsica | M.Herrmann |
| RS5335 | rotten potato | Germany (south) | M.Herrmann |
| RS5336 | rotten potato | Germany (south) | M.Herrmann |
| RS5337 | rotten potato | Germany (south) | M.Herrmann |
| RS5498 | organic compost | US (East), California,Salinas | H. Ferris |
| RS5499 | *Leptinotarsa decemlineata* (Chrysomelidae) | US (East), Ohio | C.Hoy |
| RS5500 | *Leptinotarsa decemlineata* (Chrysomelidae) | US (East), Bridgewater, (ME) | A.Miller |
| RS5501 | *Leptinotarsa decemlineata* (Chrysomelidae) | US, Madison (H61), (WI) | A.Miller |
| RS5502 | *Leptinotarsa decemlineata* (Chrysomelidae) | US (East), Freyburg (ME) | A.Miller |
| RS5503 | *Leptinotarsa decemlineata* (Chrysomelidae) | US, Madison (H01), (WI) | A.Miller |
| RS5504 | *Leptinotarsa decemlineata* (Chrysomelidae) | US (East), Bridgewater, (ME) | A.Miller |
| **(continued) Supplementary table 1** *Pristionchus uniformis* strains used in the study | | | |
| Strain | Host taxon/soil | sampling location | collector |
| RS5505 | *Leptinotarsa decemlineata* (Chrysomelidae) | US (East), East New Market, (MD) | A.Miller |
| RS5506 | *Leptinotarsa decemlineata* (Chrysomelidae) | US (East), Madison (HAES), (WI) | A.Miller |
| RS5507 | *Leptinotarsa decemlineata* (Chrysomelidae) | US (East), Bridgewater, (ME) | A.Miller |
| RS5508 | *Leptinotarsa decemlineata* (Chrysomelidae) | US (East), Ohio | C.Hoy |
| RS5509 | *Leptinotarsa decemlineata* (Chrysomelidae) | US, Madison (H01), (WI) | A.Miller |
| RS5510 | *Leptinotarsa decemlineata* (Chrysomelidae) | US (East), Freyburg (ME) | A.Miller |
| RS5511 | *Leptinotarsa decemlineata* (Chrysomelidae) | Germany (south), Tübingen | A.Weller |
| SB6016 | soil | Corsica | H. Schulz-Key |

| **Supplementary table 2**  Comparison of life history traits among representative strains | | | |
| --- | --- | --- | --- |
| *P. uniformis* strain | host taxon/soil | sampling location | larval development (h) |
| RS5070 | *Melolontha melolontha* (Scarabaeidae) | US | 72 |
| RS5244 | *Leptinotarsa decemlineata* (Chrysomelidae) | Germany | 74 |
| RS5303 | *Phyllophaga anxia* (Scarabaeidae) | US | 72 |
|  |  |  |  |
|  |  |  | brood size |
| JT11544 | rotten tomato | US | 93 |
| RS0141 | *Melolontha melolontha* (Scarabaeidae) | Germany | 91 |
| RS5070 | *Melolontha melolontha* (Scarabaeidae) | Germany | 116 |
| RS5244 | *Leptinotarsa decemlineata* (Chrysomelidae) | US | 156 |
| RS5245 | *Amphimallon* sp. (Scarabaeidae) | Serbia | 170 |
| RS5248 | *Leptinotarsa decemlineata* (Chrysomelidae) | Germany | 124 |
| RS5254 | soil | Montenegro | 112 |
| RS5285 | *Carabidae* sp. | Bulgaria | 137 |
| RS5287 | *Phyllopertha* (Scarabaeidae) | Germany | 122 |
| RS5303 | *Phyllophaga anxia* (Scarabaeidae) | US | 137 |
| RS5322 | *Leptinotarsa decemlineata* (Chrysomelidae) | Germany | 101 |
| Legend: larval development represents the total amount of hours to develop from egg to last larval stage (L4). Brood size is defined by the average amount of living offspring. | | | |

| **Supplementary table 3**. *nd2* mitochondrial gene haplotype variation and distribution among hosts |  |  | | |  | |  |
| --- | --- | --- | --- | --- | --- | --- | --- |
| Position and base composition of informative sites | Number of each haplotype associated with specific host | | | | | | |
| 111 1111111111 1111111111 1111111111 1111111111 11111111  111 1111112222 2222223333 3333344444 4455555555 5566666667 7777777778 8888888889 9999999000 0000011111 1111222222 2222333333 3334444444 44555555  1345789012 3457890123 4567890123 5678901367 8901234567 8912467890 1234567890 1234567890 2345789023 4568901234 5789012345 6789123456 7891234567 89012345 | haplotype | | | host 1 | host 2 | other | |
| GTAAGTTCTT AATGAAGTCG GCACAATTAT AAGAACAACA AACATTGTAT TTATTGGTTA CATTTTGAAA TTTTTTTGCA ACTAATAATG CGATCGTAAT AAACCCAAGA TTTACACTAT AGGTGGGAAG TGAATGTC  .......... .......... .......... .......... .......... .......... .......... .......... .......... .......... .......... .......... .......... ........  .......... .......... .......C.. .....T.... .......... ....C..... .......... .......... .......... .......... ..G....... .......C.. ....A..... ........  .......... .......... .......... .....T.... .......... .......... .......... .......... .......... .......... ..G....... .......... ....A..... .......T  ....A..... .......... T......... .....T..T. .......... .......... .......... .......... .......... ....T..... ..G....... .......... ....A..... ........  .......... .......... .......... .....T.... .......... .......... .......... .......... .......... .......... .......... .......... ....A..... C.......  .......... .......... .......... .......... .......... .......... .......... .......... .......... .......... .......... .......... .......... ........  .GT.A.AT.. .GCA.T.AT. ATGT.G..G. G.A.TT.GTG GTT...AC.C .....A..G. ..CCCCA... ......C.T. TTAGTA.GCA TAG.T..TGC G.TTTGGT.T ..A.T.TA.C GATCAAAT.A ...G.A.A  .......... .......... .......... .....T.... .......... .......... .......... .......... .......... .......... .......... .......... ....A..... C.......  .....C.... .......... .......... .....T.... .......... .......... .......... ......C... .......... ....T..... ..G....... ..C....... ....A..... ........  A.TT...T.. T..A.T.GTA ATGT.G..G. ..A.TT..T. .C..ACAC.. ......T... T......T.G C......ATG TT.......A TAG.TA.... ..TTTA.T.T AACTTGTG.. .TT.AA.T.A .AGGC.AG  .......... .......... .......... .....T.... .......... .......... .......... .......... .......... .......... .......... .......... ....A..... C.......  A.TT...T.. T..A.T.GTA ATGT.G..G. ..A.TT..T. .T..ACAC.. ......T... T......T.G .......ATG TT.......A TAG.TA.... ..TTTA.T.T AACTTGTG.. .TT.AA.T.A .AGGC.AA  ....A..... .......... T......... .....T..T. .......... .......... .......... .......... .......... .......... .......... .......... ....A..... ........  A.TTA..T.. T..A.T.ATA ATGT.G..G. ..A.TT..T. .T..G.A.G. ......T..T T......TGG ......CATG TT.......A TA..TA.... ..TTTA.T.T AACTTGTG.. GTT.AA.T.A .AGGC.AG  A.TT.C.T.. T..A.T.GTA ATGT.G..G. ..A.TT..T. .T..ACA... ......T... T......T.G .......ATG TT.......A TAG.TA.... ..TTTA.T.T AACTTGTG.. .TT.AA.T.A .AGG..AA  A.TTA..T.. T..A.T.ATA ATGT.G..G. ..A.TT..T. .T..A.A... ......T..T T......TGG ......CATG TT.......A TA.CTA.... ..TTTA.T.T AACTTGTG.. GTT.AA.T.A .AGGC.AG  .......... ....G..... .......... .....T.... .......... .C........ .......... C......... .......... .......... ..G....... ....T..... ....A..... ........  A.TTA..T.. T....T.ATA ATGTGGG..C ..A.T...T. .TTGG.A... C.GC..TA.. T......T.G .......ATT TT..C.G..A TAG.TAC... ..TTTA.T.T AACTTGTGG. .TT.AA.TGA .AGG.A.A  .CT.A..TC. .G..GT.ATA ATGT.GC..C .TAGT..G.. .TTG..A... ...C.TT... TG........ .ACC...AT. TT.....G.A TAGCTAC... ..CTTA.C.G .A..T..A.C ..C..A.T.. ..GG...T  .CT.A..T.. .G..GT.ATA ATGT.GC..C .TAGT..G.. .TTG..A... ...C.TT... TG........ .ACCC..AT. TT.....G.A TAGCTAC... ..CTTA.T.G .A..T..A.C ..C..A.T.. ..GG...T  .CT.A..TC. .G..GT.ATA ATGT.GC..C .TAGT..G.. .TTG..A... ...C.TT... TG........ .ACC...AT. TT.....G.A TAGCTAC... ..CTTA.C.G .AC.T..A.C ..C..A.T.. ..GG...T  .CT.A..TC. .G..GT.ATA ATGT.GC..C .TAGT..... .TTG..A... ...C.TT... TG........ .ACC...AT. TT.....G.A TAGCTAC... ..CTTA.C.G .AC.T..A.C ..C..A.T.. ..GG...T  .......... .......... .......... .......... .......... .......... .......... .......... .......... .......... .......... .......... .......... ........  .......... ........T. .......C.. .....T.... .......... .......... .......... .......... .......... .......... ..G....... .......C.. ....A..... ........  .......... .......... .......... .......... .......... .......... .......... .......... .......... .......... .......... .......... .......... ........  .......... .......... .......... .......... .......... .......... .......... .......... .......... .......... .......... .......... .......... ........  A......... .......... .......... .....T.... .......... .......... .......... .......... .......... .......... .......... .......... ....A..... C.......  .......... .......... ........T. .....T.... .......... .C........ .......... C......... .......... .......... ..G....... .......... ....A..... ........  AAT.A..T.C .G.AGT.AT. ATGTGG..G. G.A.TTGGTG GTT..CAC.C .....A..G. T....C.G.. .C...CC.T. TTAGTA.GCA TAG.TA.T.. GGTTTGGTAT ..A.T.TA.C ..C.AA.T.A ..GG.A.G  .......... .......... .......... .......... .......... .......... .......... .......A.. .......... .......... .......... .......... .......... ........  .GT.A.AT.. .GCA.T.AT. ATGT.G..G. G.A.TT.GTG GTT...AC.C .....A..G. ..CCCCA... ......C.T. TTAGTA.GCA TAG.T..TGC G.TTTGGT.T ..A.T.TA.C .ATCAAAT.A ...G.A.A  .C..A..TC. ...A..A.TA A.......GC G..GT...T. .TTG..A... ......T... T........G ..A.C..AT. .T....G... .A..TA.... ..TTTT.T.T ....A.TGG. ..A.A....A .A...A.T  .....C.... .......... ....C..... .....T.... .......... .......... .......... ......C... .......... ....T..... ..G....... ..C....... ....A..... ........  .......... .......... .......... .......... .......... .......... .......... .......... .......... .......... .......... .......... .......... ........  .GT.A..TC. .G.A.T.AT. ATGT.GC.G. G.A.T..G.G GTT..CAC.C ..G..AT.A. .G...C.G.. .....C..T. TTAGTA.G.A .AG.T..T.C GGTTTGGTAT ..A...TA.C .ATCAA.T.A ..GG.ACA  .....C.... .......... .......... .......... .......... .......... .......... .......... .......... .......... .......... .......... .......... ........  .C........ ........T. .......... .....T.... .......... .......... T......... .......... ....T..... .......... .......... .......... G...A..... ........  .C........ ........T. .......... .....T.... .......... .......... T......... .......... .......... .......... .......... .......... G...A..... ........  A.TTA..T.. T...GT.A.A ATGTGGG... ..A.TT..T. .TT.AC.... C.G...TA.. T......T.G .C.....ATG TT..T.G..A TA..TA.... T.TTTA.T.T AACT.GTG.. .TT.AA.TGA CAGG.A.G  .......... .......... .......C.. .....T.... .......... ....C.T... .......... .......... .......... .......... G.G....... .......... ....A..... ........  AAT.A..T.C .G.AGT.AT. ATGTGG..G. G.A.TTGGTG GTT...ACGC .....A..G. .......... .CC..CC.T. TTAGTA.GCA TAG.TA.T.. GGTTTGGTAT ..A.TGTA.C ..C.AA.T.A ..GG.A.G  ....A..... .......... T......... .....T..T. .......... .......... .......... .......... .......... .......... .......... .......... ....A..... ........  ....A..... .......... T......... .....T..T. .......... .......... .......... .......... .........A .......... .......... .......... ....A..... ........  .......... .......... .T........ .....T.... .......... .......... .......... .......... .......... .......... ..G....... .......... ....A..... .....A..  .......... .......... .......... .....T.... .......... .......... .......... .......... .......... .......... ..G....... .......... ....A..... ........  .......... .......... .......... .....T.... .......... .......... .......... .......... .......... .......... ..G....... .......... ....A..... ........  .......... .....GA... .......... .....T.... ...G...... .C........ .......... .......... .......... .......... ..G....... .......... ..A.A..... ........  .......... ....G..... .......... .....T.... .......... .C........ .G........ C......... .......... T......... ..G....... ....T..... ....A..... ........ | Hap_1*****  Hap_2*****  Hap_3*****  Hap_4*****  Hap_5*  Hap_6*****  Hap_7  Hap_8  Hap_9  Hap_10  Hap_11  Hap_12  Hap_13*****  Hap_14  Hap_15  Hap_16  Hap_17  Hap_18  Hap_19  Hap_20  Hap_21  Hap_22  Hap_23  Hap_24  Hap_25  Hap_26  Hap_27  Hap_28  Hap_29  Hap_30  Hap_31  Hap_32  Hap_33  Hap_34  Hap_35  Hap_36  Hap_37*****  Hap_38  Hap_39  Hap_40  Hap_41  Hap_42  Hap_43  Hap_44  Hap_45  Hap_46  Hap_47  Hap_48  Hap_49 | | | 1  2  1  1  1  2  0  0  0  0  0  0  1  0  1  1  1  0  0  1  1  1  1  0  0  0  1  0  0  0  0  1  0  0  1  0  1  0  0  0  1  0  0  0  0  0  0  0  0 | 1  1  3  2  1  5  1  1  1  1  0  1  1  1  0  0  0  3  0  0  0  0  0  0  0  1  0  0  0  0  0  0  0  0  0  0  1  0  0  0  0  3  1  0  0  1  1  1  0 | 0  1  1  1  0  0  0  3  0  0  1  1  0  0  0  0  0  1  1  0  0  0  0  1  1  2  0  1  1  1  1  0  1  1  0  1  1  1  1  1  0  0  0  1  1  0  0  0  1 | |
| (continue) | | | | | | | |
| **(continued) Supplementary table 3** |  | | | | | | |
| Position and base composition of informative sites | Number of each haplotype associated with specific host | | | | | | |
| 1111111111 1111111111 1111111111 1111111122 2222222222 2222222222 2222222222 2222222222 2222222222 2222222222 2222222222 2222222222 3333333333 333  5556666666 6777777778 8888888899 9999999900 0000000111 1111222222 2333333444 4444455555 5555666666 6667777777 7788888888 8999999999 0000001111 111  7890123456 8012456780 1245678901 2345678902 3456789012 4579013456 9025789012 3467901235 6789012345 6791234567 8912345678 9012456789 0134781345 679 | haplotype | | host 1 | | host 2 | other | |
| GTTTAATTTA GATATTATGT TATACACGGA AGTTACTTTT ATAATTTTGT TGGTAGACTT TCGTGAGATG GTATTTAAAG GTAAACTATA TAACATAGCT TGTAACAAGT TATAGTCAAA AATATCAGTA ATA  .......... .......... .......... .......... .......... .......... .......... .......... .......... .......... .......... .......... .......A.. ...  .......... .......... .......... ......C... .......... .......... .......... .......... .......... .......... .......... .......... .......A.. ...  .......... ........A. .......... .......... .......... .......... .......... .......... .......... .......... .......... ..C....... .......A.. G..  ..C....... .......... .......... .......... .......... .......... ......A... .......... .......... .......... .......... .......... .......A.. ...  .......... .......... .......... .......... .......... .......... ........A. .......... ....G..... .......... .......... .G........ .......A.. ...  .......... .......... .......... .......... .......... .......... .......... .......... .......... .......... .......... .......... .......A.. ...  ...CGG.C.. AGCC..TAA. CGATT.TAAG GACATTA.C. .....C.... .A.CGA.T.. ATT.AGA..A T.....T.GA TC.T.T.G.G ..GA.A.A.. ...G.TGTAC ....ACTT.T .G..CT.ACG ...  .......... .......... .......... .......... .......... .......... ........A. .......... ....G..... .......... .......... .G........ .......A.. ...  .......... .......... .......... .......... .......... ....T..... .T........ .......... .......... .......... .......... .......... .......A.. ...  .C........ T..TC.TAA. ..ATT.TAA. .......... .C.GG.C.A. ....GA.ACG .TTAA..... TC.CC...GT T.G..T.... C..TT..A.A CA...T.... ...G....G. G....TGAC. ...  .......... .......... .......... .......... .......... .......... .T......A. .......... ....G..... .......... .......... .G........ .......A.. ...  .C........ ...TC.TAA. ..ATT.TAA. .......... .C.GG.C.A. ....GA.ACG .TTAA..... TC.CC...GT T.G..T.... C..TT..A.A CA...T.... ...G....G. G....TGAC. ...  ..C....... .......... .......... .......... .......... .......... ......A... .......... .......... .......... .......... .......... .......A.. ...  AC.......G ...TC.TAA. ..ATT.TAA. .......... .C.GA.CCA. ....GA.A.G .TTAA.A... T...C...GT T.G..T.... C..TT..A.A CA..GT.... ........G. G....TGA.. ..G  .C........ ...TC.TAA. ..ATT.TAA. .......... .C.GG.C.A. ....GA.ACG .TTAA..... TC.CC...GT T.G..T.... C..TT..A.A CA...T.... ...G....G. G....TGAC. ...  AC.......G ...TC.TAA. ..ATT.TAA. .......... .C.GA.CCA. ....GA.A.G .TTAA..... T...C...GT T.G..T.... C..TT..A.A CA..GT.... ........G. G....TGA.. ..G  .......... .......... .......... .......... .......... .......... .......... .......... .......... .......... .......... .......... .......A.. G..  ....GG.... A..TCCTAA. ..ATT.TAA. G......... G..GG.C... .....A.T.G .TTAA..G.. TC......GT T....T.... .G.T...T.A .A...T.... .......... ..C..T.... .CG  ...CGGC... A..T.CTAAC ..ATTGTAA. .......... ..G...C.A. CAA.G..G.A CTTG..A..A TC..A..G.T A...G...C. CG.T.C.A.C .A.G.TGG.. CGC....... ......GA.. ..G  ...CGGC... A..T.CTA.C ..ATTGTAA. .......... ..G...C.A. CAA....G.A CTTG..A..A TC..A..G.T A...G...C. CG.T.C.A.C .A.G.TGG.. .GC....... ......GA.. ..G  ...CGGC... A..T.CTAAC ..ATTGTAA. .......... ..G...C.A. CAA.G..G.A CTTG..A..A TC..A..G.T A.......C. CG.T.C.A.C .A.G.TGG.. CGC....... ......GA.. ..G  ...CGGC... A..T.CTAAC ..ATTGTAA. .......... ..G...C.A. CAA.G..G.A CTTG..A..A TC..A..G.T A...G...C. CG.T.C.A.C .A.G.TGG.. CGC....... ......GA.. ..G  .......... .......... .......... .......... .......... .......... .T........ .......... .......... .......... .......... .......... .......A.. ...  .......... .......... .......... ......C... .......... .......... .......... .......... .......... .......... .......... .......... .......A.. ...  .......... .......... .......... .......... .......... .......... .T........ .......... .......... .......... .......... .......... .......A.. ...  .......... .......... .......... .......... .......... .......... .......... .......... ......C... .......... ......G... .......... .......A.. ...  .......... .......... .......... .......... .......... .......... .T......A. .......... ....G..... .......... .......... .G........ .......A.. ...  .......... .......... .......... .......... .......... .......... .......... .....C.... .......... .......... .......... .......... .......A.. G..  A..CGG.CC. ...T...AAC C.ATT.TAA. .A....AC.C ......C.AA .A.CGAGTC. ATT.A.A..A T.T.C.G..A ....G...C. C....C.A.. C.C..T.TAC .G.G...... G..G.T.A.. .A.  .......... .......... .......... .......... .......... .......... .......... .......... .......... .......... .......... .......... .......A.. ...  ...CGG.C.. AGCC..TAA. CGATT.TAAG GACATTA.C. .....C.... .A.CGA.T.. ATT.AGA..A T.....T.GA TC.T.T.G.G ..GA.A.A.. ...G.TGTAC ....ACTT.T .G..CT.ACG ...  A......... ........A. ..AT..T... .AC....... ........A. .......TCG .TAGA..... A.....T... A.......C. ......GA.. .A..GT.... A......... ...G...A.. ..G  .......... .......... .......... .......... .......... ....T..... .T........ .......... .......... .......... .......... .......... .......A.. ...  .......... .......... .......... .......... .......... .......... .......... .......... ......C... .......... ......G... .......... .......A.. ...  A..C...C.. ..CC...AA. ..ATT.TAAG .ACAT.A.C. ......C.AG .A...A.... ATT......A T....CT.G. T..T.T.G.. C.GAGA.... .A..GTGTAC ....A.TT.T .G...T.AC. .A.  .......... .......... .......... .......... .......... .......... .......... .......... .......... .......... .......... .......... .......A.. ...  AC.......G .......... .........G .......... .......... .......... .......... .......... .......... C.......T. ....GT.... .......... .......A.. ...  AC.......G .......... .........G .......... .......... .......... .......... .......... .......... C.......T. ....GT.... .......... .......A.. ...  ....G..... ...T..TAA. ..ATT.TAA. .........C G..GA..CAC .....A.T.G CTTAA..GC. T...C...GT T......... ...T...T.A .ACG.T.... .......... G.C..T.A.G .CG  .......... .......... .......... .......... .......... .......... .......... .......... .......... .......... .......... .......... .......A.. ...  ...CGG.CC. ...T...AAC C.ATT.TAA. .A....AC.C ......C.AA .A.CGAG.C. A.T.A.A..A T.T.C.G..A ....G...C. C....C.A.. ..C..TGTAC ...G...... ...G.T.AC. .A.  ..C....... .......... .......... .......... .......... .......... ......A... .......... .......... ......G... .......... .......... .......A.. ...  ..C....... .......... .......... .......... .......... .......... ......A... .......... .......... .......... .......... .......... .......A.. ...  .......... .......... ....T..... .......... .......... .......... .......... .......... .......... .......... .......... .......... .......A.. ...  .......... .......... ....T..... .......... .......... .......... .......... .......... .......... .......... .......... .......... .......A.. ...  .......... T......... .......... .......... .......... .......... .T........ .......... .......... ........T. .......... .......... .......A.. ...  .......... .......... ....T..... .......... .......... .......T.. .T........ .......... .......... .......... .......... .......... .......A.. ...  .......... .......... .......... .......... .......... .......... .......... .......... .......... .......... .......... .......... .......A.. G.. | Hap_1*****  Hap_2*****  Hap_3*****  Hap_4*****  Hap_5*  Hap_6*****  Hap_7  Hap_8  Hap_9  Hap_10  Hap_11  Hap_12  Hap_13*****  Hap_14  Hap_15  Hap_16  Hap_17  Hap_18  Hap_19  Hap_20  Hap_21  Hap_22  Hap_23  Hap_24  Hap_25  Hap_26  Hap_27  Hap_28  Hap_29  Hap_30  Hap_31  Hap_32  Hap_33  Hap_34  Hap_35  Hap_36  Hap_37*****  Hap_38  Hap_39  Hap_40  Hap_41  Hap_42  Hap_43  Hap_44  Hap_45  Hap_46  Hap_47  Hap_48  Hap_49 | | 1  2  1  1  1  2  0  0  0  0  0  0  1  0  1  1  1  0  0  1  1  1  1  0  0  0  1  0  0  0  0  1  0  0  1  0  1  0  0  0  1  0  0  0  0  0  0  0  0 | | 1  1  3  2  1  5  1  1  1  1  0  1  1  1  0  0  0  3  0  0  0  0  0  0  0  1  0  0  0  0  0  0  0  0  0  0  1  0  0  0  0  3  1  0  0  1  1  1  0 | 0  1  1  1  0  0  0  3  0  0  1  1  0  0  0  0  0  1  1  0  0  0  0  1  1  2  0  1  1  1  1  0  1  1  0  1  1  1  1  1  0  0  0  1  1  0  0  0  1 | |
| Positions of variable sites refer to nucleotide positions relative to the 789 bp mt gene *nd2* sequences. Host 1and host 2 refers to scarab spp and *Leptinotarsa decemlineata* respectively. Other refers to haplotypes collected from soil or non-scarab or non-*L. decemlineata* host. Asterisks indicate shared haplotype between scarab spp. and  *L. decemlineata* | | | | | | | |

| **Supplementary table 4** Divergence estimates of mitochondrial genes in different *Pristionchus* species in *cyt b* | | | | | | | |
| --- | --- | --- | --- | --- | --- | --- | --- |
|  | *n* | S | H | Hd | π | θ | Tajima’s D |
| *P. uniformis** | 62 | 147 | 32 | 0.949 | 0.064 | 0.072 | 0.190 |
| *P. aerivorus** | 5 | 71 | 5 | 1.000 | 0.067 | 0.067 | 0.849 |
| *P. maupasi*** | 23 | 11 | 12 | 0.893 | 0.055 | 0.040 | 2.00 |
| *P. pacificus*** | 22 | 71 | 19 | 0.983 | 0.032 | 0.043 | -0.636 |
| *P. entomophagus*** | 20 | 40 | 10 | 0.837 | 0.015 | 0.021 | -1.032 |
| Legend: S, number segregating sites; H, number of haplotypes; Hd, haplotype diversity; π, nucleotide diversity; θ, level of polymorphisms from S; Tajima’s D test for the assumption of neutral sequence selection evolution. Data for *P. pacificus* are from Zauner *et al.* 2007 and Molnar *et al.* 2011. *: gonochoristic species, **: hermaphroditic species. | | | | | | | |
